# A cross-cultural, cross-disciplinary, and cross-gender study on Appraisal resources in PhD dissertation abstracts: Martin & White's (2005) Appraisal Theory in focus

**DOI:** 10.1016/j.heliyon.2023.e22074

**Published:** 2023-11-06

**Authors:** Ali Hashemi, Fatemeh Mahdavirad

**Affiliations:** English Language and Literature Dept., Faculty of Language and Literature, Yazd University, Yazd, Iran

**Keywords:** PhD thesis, Appraisal theory, Abstract, Native/non-native writer, Male/female writer, Soft/hard science

## Abstract

The abstract which complements proposals, articles, and dissertations, is a remarkable convention in scientific studies since it creates access for readers and authors to read or publish studies or articles. Research abstracts (RA) function as the gateway to view an article, journals' selection for contributions, and for conferences to accept or reject articles (Lores, 2004) In this study, we aimed to investigate the preferences of writers in 160 PhD dissertation abstracts, encompassing both male and female native and non-native authors, across various fields of study, both in the hard and soft sciences. Our primary objective was to discern the writers' inclinations towards utilizing specific linguistic resources, as proposed by Martin and White's Appraisal theory (2005), to convey their positions and engage with the perspectives articulated by their peers. The analysis of the data, conducted using statistical methods, unveiled a pervasive utilization of appraisal resources by the writers, enabling them to articulate their viewpoints, prospects, perceptions, and evaluations concerning diverse subjects. Among these resources, Attitude resources stood out prominently, constituting a substantial 84% of the total Appraisal resources employed in all the abstracts. Graduation resources held an intermediate position, while Engagement resources were the least utilized. Within the realm of Attitude subcategories, Appreciation resources emerged as the most prevalent. Remarkably, female authors specializing in the soft sciences displayed a higher degree of proficiency in the use of these resources, surpassing their counterparts in other categories. This finding suggests that female writers in the soft sciences possess exceptional interpersonal communication skills, making them particularly persuasive and inspirational. The implications of this study extend to the domains of language teaching and learning, material development, and syllabus design. It sheds light on how writers employ linguistic resources to convey their positions effectively, offering valuable insights for educational practices and curriculum enhancement.

## 1 Introduction

Scientific studies and academic research play a crucial role in advancing knowledge and understanding across various disciplines [[41], [42], [45], [46]]. These endeavors provide a systematic and rigorous approach to investigating research questions, testing hypotheses, and generating new insights [39]. By employing well-defined methodologies and adhering to rigorous ethical standards, researchers aim to ensure the reliability and validity of their findings [44].

The dissemination of research findings is an essential aspect of scientific studies and academic research. Scholarly publications, including journals, conference proceedings, and academic books, serve as the primary means of sharing research outcomes with the wider academic community [33]. These publications typically undergo a rigorous peer-review process, involving independent experts in the field who assess the quality and rigor of the research [38].

Within the realm of academic research, doctoral dissertations hold a special significance. A PhD dissertation represents the culmination of several years of dedicated study and research in a specific area [36]. Dissertations are rigorous academic documents that contribute to the existing body of knowledge by exploring research problems, conducting original research, and presenting a comprehensive analysis of findings [35].

Abstracts, an integral component of scientific studies, serve as concise summaries of research articles, proposals, and dissertations. They play a crucial role in facilitating access to research for both readers and authors [1]. Research abstracts function as gateways for readers to assess the relevance and significance of the research, while they also assist journals and conferences in the selection and evaluation of contributions [50].

The abstract section of a research article plays a crucial role in conveying the essence of the study to readers. It serves as a concise summary that highlights the research's significance, objectives, key findings, and background. Crafting an effective abstract is essential for researchers to demonstrate the relevance of their work, contribute to the existing knowledge, and increase the likelihood of acceptance in prestigious academic journals. Consequently, abstracts have gained increasing prominence in scholarly communication.

Abstracts, often freely accessible online, provide a gateway for readers to access articles, aid journal editors in selecting contributions, and assist conference organizers in evaluating submissions [1]. The ability to write informative and engaging abstracts has become indispensable for academic scholars. Analyzing the structure, organization, and language of abstracts can offer valuable insights for researchers seeking to enhance their abstract writing skills.

Abstracts are a distinct genre of discourse aimed at discussing new knowledge within various scientific disciplines [2]. They serve as a crucial tool for specialized reading, enabling readers to assess the relevance of a study to their own research goals and providing the necessary background to determine if a particular article deserves closer attention [[3], [4], [5]]. Moreover, abstracts are considered time-saving devices, providing concise and precise information about the content and indicating whether an article merits further exploration [6].

Scholars hold differing views on the functions of abstracts. Swales [7,48] suggests that abstracts serve as both summaries and purified reflections of the entire article, while Bhatia [8] emphasizes their informative function, stating that abstracts offer a faithful and precise summary that represents the entire article. Abstracts also serve as decision-making points for readers, helping them determine whether the full article warrants closer scrutiny [9]. Consequently, academics strategically employ rhetorical features in abstracts to engage readers and encourage them to delve into the complete article [10]. The abstract section represents readers' first encounter with the research, and the majority of academic journals require authors to submit an abstract alongside their original papers [5,11].

Some critics, however, oppose the functionality of abstracts and have counterargument. They (e.g., [[34], [47]]) argue that abstracts oversimplify complex research studies, potentially leading to a loss of nuance and essential details. They claim that the limited space and concise nature of abstracts may not adequately capture the intricacies and depth of the research findings.

Others contend that abstracts may exhibit a bias towards positive or significant results, potentially leading to publication bias and the exclusion of negative or non-significant findings [[37], [40]]. This criticism suggests that abstracts may not always present a comprehensive picture of the research, creating a skewed perception of the overall body of knowledge.

This study, however, attempts to put forth, by examining numerous abstracts, key features that contribute to a well-crafted abstract. Such features include conciseness, simplicity, and precision, clearly articulating the hypothesis and objectives while avoiding excessive experimental details and statistical methods. An effective abstract provides a snapshot-like view of the entire article, succinctly conveying the main findings [5].

## 2 Literature review

Understanding how scholars employ appraisal resources in dissertation abstracts can offer insights into the strategies employed to position themselves in their respective fields and convey the significance and novelty of their research [49]. Moreover, this analysis may have implications for language teaching, material development, and syllabus design [43]. By investigating the use of appraisal resources in the abstracts of PhD dissertations, this study contributes to the broader understanding of academic writing practices and their potential impact on scholarly communication.

The abstract section is the main part of the research article to present why the author has explored the topic. It is also the first viewpoint for readers to know about the problems of the topic that will be answered in the sections of the research articles. The most important reasons for including an abstract into an article are *selection* and *indexing*. As for the selection, by reading the abstract introduction, readers can infer the reasons why the writer takes the topic to be investigated. Thus, abstracts allow readers who may be interested in the longer work to quickly decide whether it is worth their time to read it. In addition to this, indexing is another major reason for writing an abstract as many online databases use abstracts to index larger works. Therefore, abstracts should contain keywords and phrases that allow for easy searching.

Generally speaking, Walter [12,51] defines abstract as a shortened form of a speech, article, book, etc., giving only the most important facts or ideas. In academic world as defined by Bhatia [8]; the word abstract means, a description or factual summary of the much longer report, and is meant to give the reader a precise and concise knowledge of the full article. The generic purpose of research article abstracts, according to Martín [13]; is to provide the summary of the content of the accompanying article. Consequently, he suggests the writers of the research article (RA) abstract to present an abstract in a conventionalized form by using a series of rhetorical strategies or move structures.

### 2.1 The importance of article abstracts

Earlier studies have given important roles to research article abstracts for the purpose of informing the reader about the content of the article by condensing the most important findings in its writing. It also allows the reader to decide whether to read the entire article or not [4]. Thus, the abstract, alongside the title, is a vital issue in deciding whether the whole article will be read. The abstract, functioning as the summary of the study, should be expressive, to give the reader an idea of the focus of the study. Accordingly, most readers first review the abstract and then decide to read the entire article based on their first view and impression of it.

According to Berkenkotter & Huckin [14]; there are four reasons that make abstract play an important role in research articles. First, it provides important information or statements that are easy to be accessed. Second, it functions as the screening device that can help readers to decide whether they will finish reading the whole content. Third, it gives a framework for readers to read the article. Fourth, it provides summaries of primary points of a research article.

Abstracts help the writer to bunch the most important and significant findings into a small number of words. This could save the reader's time as it informs the reader about the exact content of the article, indicating whether the full text merits their further attention [11]. Abstracts also help the writer to convince conference organizers or journal editors to accept or publish the article. Having these multiple functions in mind, textual analysis of the abstract as a genre could provide the reader and the writer with helpful insights concerning its underlying macro (rhetorical) and micro (linguistic features) structures [1].

Writing the abstract part of a research article usually involves the interpersonal voice and reasons for creating a sense of persuasion that will attract readers. A quality abstract research article can be accomplished by using appraisal resources to represent the writers' ideas and propositions effectively. Moreover, a research article abstract involves the writers' personal voice in exploring the topic and this is needed to help readers enrich their knowledge.

In writing a thesis, the abstract is considered as an important part that will bring readers to read the content [15], and the first section to be read by examiners [16]. Readers or examiners of research articles or papers are busy people who have lots of works to do. For that reason, most readers limit their initial research looking at titles and abstracts before choosing what Research Articles (RA) to select and read [17].

Composing an abstract is a demanding task and, based on the literature, novice writers find it difficult to construct well-structured and appropriate abstracts. Busch-Lauer [18]; for instance, noticed some structural and linguistic shortcomings in English abstracts written by Germans, which, accordingly, might impede the general readability for the scientiﬁc community. Writing an abstract requires knowledge of the genre and conventions and procedures for creating an appealing, convincing, and a forceful abstract to trigger the interest and curiosity of the readers, publishers, reviewers, and examiners and increase the likelihood of its acceptance. In addition, creating a good research article abstract necessitates a primary concentration of rhetorical and textual features that establish an effective research article abstract.

### 2.2 Previous studies on abstracts

Scholars recognize research article abstracts as a specific genre. Hyland (2004a) defines the genre as a term for grouping texts together, representing how writers typically use language to respond to recurring situations. Furthermore, Martin (cited in Ref. [19] holds that a genre is a purposeful goal-oriented activity in which writers or speakers are involved as members of our community. Therefore, the recognition of the genre of a text plays an imperative part in identifying ways in which a particular piece of writing resembles to, or is indicative of, other texts published in the field [19]. Eggins [19]; additionally, states that in case a text does not easily fit a genre, then it is in some ways a problematic text. Accordingly, as Martín [13] holds, a major focus of attention in analyzing a specific genre has been the investigation of the organizational patterns of English research articles.

Many researchers have been studying the rhetorical structures, linguistic features, and generic moves of research article abstracts in their studies. Analyzing the influence of the choice of language on the abstracts of different fields, Melander et al. [20] found differences in the general structure of the abstracts of linguistics and biology in the USA. In another study, Stotesbury [21] found that evaluation was two times more frequent in the abstracts written in humanities and social sciences than those written in the basic sciences. Doing abstract genre analysis, Huckin [22] noticed that the abstracts written in biomedical article often lack the purpose move, and in her 2002 study, Samraj [32]. realized that the central argument moves in Conservation Biology are more critical as compared to Wildlife Behavior.

Some of these variations and changes in structure and rhetorical features are language and culture specific. Analyzing the effect of language on abstracts in the US and Sweden, Melander et al. [20] found cultural and national variations in abstracts in linguistics realizing that there are differences in the general structure of linguistics and biology abstracts. In a 2003 study of cultural variations in the rhetorical structure of social science abstracts, Martin-Martin noticed a preference to ignore the Results part in Spanish authors while this is a quite frequent feature of English abstracts. These variations, Martin-Martin [4] concludes, indicate the social connections between writers and readers in diverse cultures and discourse communities. In another study of cross-cultural differences in Spanish and English abstracts, Martin-Martin and Burgess [23] noticed that English abstracts favor criticism more than the Spanish abstracts. Meanwhile, while English favors impersonal and indirect criticism, Spanish is not.

Pascual & Unger [24] examined the Engagement system of the Appraisal resources in Argentinean non-native English speakers working on their proposals at the universidad Nacional de San Luis. They found that the proposals were highly heteroglossic with a variety of Engagement resources. The examples were primarily expansive, indicating that authors tend to invite rather than challenge their colleagues’ views. This may be interpreted as an attempt to address potentially diverse readers. These findings can help writers in becoming attentive of the interpersonal resources they use to position themselves and align their audiences when writing their proposals.

Martin and Thompson [25] conducted a cross-disciplinary study to analyze the use of evaluative language in research article abstracts. Drawing on Appraisal Theory, they examined a corpus of abstracts from diverse disciplines. The study identified the prevalence and patterns of attitude, graduation, and engagement resources in the abstracts. The findings provided insights into how academic writers position themselves, evaluate their research, and engage with prior scholarship in abstract writing across disciplines.

Yang and Hu [26] directed a comparative study investigating appraisal resources in thesis abstracts across different cultural contexts. Adopting Martin and White's [6] Appraisal Theory, they examined thesis abstracts from English and Chinese academic writing. The study examined the frequencies and functions of attitude, graduation, and engagement resources in the abstracts, comparing their usage across cultures. The findings shed light on how cultural differences influence the rhetorical choices and strategies employed in thesis abstracts, contributing to our understanding of cross-cultural variations in academic writing.

In another comparative study, Cao and Jiang [27] examined appraisal resources in conference abstracts. The study analyzed a corpus of abstracts from international conferences in various disciplines. By applying Martin and White's [5] Appraisal Theory, the researchers identified the types, frequencies, and functions of attitude, graduation, and engagement resources in the abstracts. The study revealed disciplinary variations in the use of appraisal resources, highlighting how scholars strategically employ evaluative language to position their research, present findings, and engage with the conference audience.

Zhang and Li [28] in their study explored appraisal resources in English and Chinese research article abstracts. Adopting Martin and White's [6] Appraisal Theory, the researchers analyzed a corpus of abstracts from English and Chinese academic journals. The study examined the frequencies and functions of attitude, graduation, and engagement resources in the abstracts, comparing their usage patterns across languages. The findings provided insights into the rhetorical strategies employed in abstract writing across different linguistic and cultural contexts, contributing to our understanding of cross-linguistic variations in academic discourse.

## 3 Methodology

### 3.1 Research corpus

This research involves an extensive analysis of a diverse and comprehensive corpus of PhD thesis abstracts. The corpus has been meticulously constructed to encompass a wide array of linguistic, disciplinary, cultural, and demographic variables. Our aim is to explore cross-cultural, cross-gender, and cross-disciplinary differences in the utilization of Appraisal resources within the abstracts of PhD dissertations.

The corpus consists of 160 PhD thesis abstracts, carefully selected to represent a rich tapestry of academic diversity. These abstracts originate from both hard and soft science fields, showcasing the linguistic variation inherent to these distinct areas of study. Additionally, the corpus reflects a balanced representation of native English writers and non-native Iranian writers, introducing cultural and linguistic variations into our analysis.

Specifically, the corpus comprises:

#### 3.1.1 Linguistic variables

• Native English and non-native Iranian writers: To examine the influence of language proficiency and cultural background on the use of Appraisal resources.
• Male and female writers: To investigate gender-based linguistic preferences and patterns.

#### 3.1.2 Disciplinary variables

Hard science and soft science fields: To capture the distinct linguistic conventions and preferences within these academic domains.

⁃ Hard science fields: Physics and mathematics.
⁃ Soft science fields: Linguistics, literature, art, psychology, and philosophy.

#### 3.1.3 Demographic variables

• A subset of 40 abstracts from non-native Iranian PhD students, offering insights into the linguistic choices of writers from diverse cultural and linguistic backgrounds.
• The remaining 120 abstracts are authored by native English speakers, serving as a point of comparison.
• In the hard science domain, 40 abstracts were selected from male writers due to limitations in finding an equal number of hard science abstracts from female writers.
• In the soft science field, 80 abstracts were chosen, encompassing contributions from both male and female authors.

By meticulously curating this corpus, we aim to conduct a thorough investigation into how linguistic, disciplinary, and cultural factors intersect and influence the use of Appraisal resources within PhD thesis abstracts. This diverse corpus provides us with a robust foundation for our analysis, allowing us to draw meaningful insights from the rich tapestry of academic discourse represented within it.

### 3.2 Analytical framework

Grounded in Halliday's [29] Systemic Functional Linguistics, Appraisal theory was developed by Martin [30]; Martin [4] and Martin and White [5]; to explore language evaluative functions, or more specifically and obviously, the semantic resources, according to Martin [30]; serving the purpose of expressing emotions, judgements and valuations, alongside the resources for intensifying and engaging with these evaluations.

Appraisal is a general term covering three groupings: Attitude, Graduation and Engagement. Attitude entails the resources applied to exchange judgements, affects, appreciations, and valuations; Engagement and Graduation, however, are concerned with the resources which engage and enhance Attitude.

**Fig. 1:**
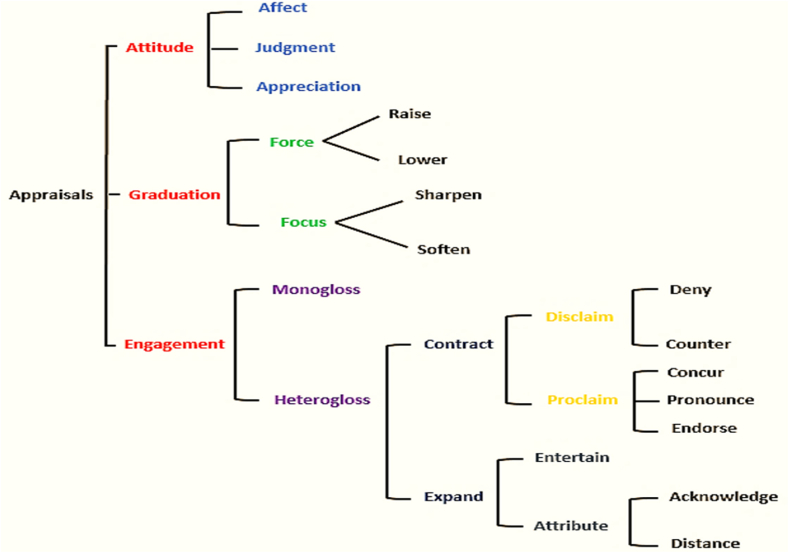
Outline of the appraisal theory [6].

Connected to the communication situation, Appraisal theory deals with the linguistic resources which writers and speakers use to express opinions towards the communicative events as well as indicate their evaluations of others' attitudes in interaction. It involves various interpersonal tools and language resources used to convey values and emotions towards propositions and the level of engagement with these positions and stances. The emphasis in Appraisal theory is, in White's [31] view, on the interpersonal meanings both within particular utterances and as the text unfolds collectively. Fig. 1 (below) outlines the Appraisal Theory.

The Appraisal Theory is quite qualified to serve as the framework to analyze the data against it. It is an extension to Halliday's [29] Functional Linguistics and has been informed by earlier developments in discourse analysis. It is a language-based theory trying systematically to determine the meanings of the utterances based on the context which is, in this sense, the task of the Tenor section of Halliday's functional grammar. Meanwhile, and more importantly, Appraisal theory incorporates opinions as distinct elements, Attitudes, and as propositions (Engagement and Graduation). Thus, unlike the traditional discourse analysis which ignored the context of communication and the level of speaker involvement in the propositions put forward by the participants, Appraisal theory is attentive to communication situation as well as the degree speaker engagement in the communicative event.

### 3.3 Instrumentation

Martin and White's [5] Appraisal Theory which is rooted in Halliday's [29] Systemic Functional Linguistics was selected as the basis for analyzing the data. Data analysis procedure involved the examination, detection, location, and quantification of Appraisal elements and discourse markers functioning to materialize writer's attitudes and propositions to the issues and prospects presented in the PhD dissertation abstracts.

The next step, the quantification stage, involved counting the Appraisal resources and calculating their frequencies and percentages. Next, in the qualitative phase, the cases of Attitude, Engagement, and Graduation resources and their subcategories, in the texts under study, were detected, categorized, coded, and assigned to relevant groupings.

For a higher level of accuracy as well as a desired level of reliability, we manually coded and double-checked all the abstract texts two times, in a two-month interval, to make sure of the consistency in data analysis and resource categorization. The results, later on, were tabulated and descriptions and explanations added to show writers’ positions and preferences for the Appraisal resources. Finally, the results were compared and contrasted for each discipline and writer groups to find out about the possible similarities and variations regarding the variables under investigation.

### 3.4 Data analysis procedure

In the current research, we employed the Appraisal theory [6] as the framework for the analysis of the data. Thus, the data were collected by detecting and recording discourse elements and markers. We, then, classified the data, assigned to relevant classes, counted, coded, and calculated the frequency and percentage of Appraisal resources. The process was checked and double checked to make sure of the reliability of the analysis in a one-month interval. The next step was the addition of figures and tables showing the distribution and frequency of the appraisal items in the abstracts. Later, descriptions and explanations were added.

The data analysis procedure for the study can be outlined as follows:

• Data Preparation involves collecting 160 PhD dissertation abstracts.
• Developing a Coding Scheme or set of categories based on the appraisal resources and their subcategories.
• Coding and Categorization involves applying the coding scheme to the abstracts to identify and mark instances of appraisal resources. This also involves assigning each instance of appraisal resource to its respective category and subcategory based on the coding scheme. Meanwhile, we made sure of consistency and accuracy in coding by double-checking and resolving any discrepancies.
• Data Analysis further into: a) Calculating the frequencies or percentages of each category and subcategory of appraisal resources in the abstracts. b) Summarizing and interpreting the overall usage of appraisal resources in terms of stances, prospects, perceptions, and evaluations of various topics. c) Identify the most prominent and least prominent categories and subcategories of appraisal resources. d) Analyzing any patterns or trends in the usage of appraisal resources.
• Interpretation and Discussion involves a) Interpreting the findings in relation to the research aim of uncovering writers' preferences for appraisal resources and their engagement with other writers' propositions and voices. b) Discussing the implications of the results for language teaching and learning, material development, and syllabus designing. c) Highlighting any limitations of the study and suggest directions for future research.

### 3.5 Rater reliability

The study involved a single rater who rated the abstracts twice with a one-month interval between the ratings. This was to make sure of the intra-rater reliability of thus consistency of the findings. This procedure made possible to eliminate the probability of any differences in rating the abstracts in the two occasions.

## 4 Results and discussion

This is a massive study of Appraisal resources in PhD dissertation abstracts used by native male and female writers in both hard and soft sciences. As we had a large corpus of abstracts and many variables, and we intended to have multiple comparisons within and across genders, cultures, and disciplines, as well as the interactive effects of these variables, the discussion, thus, will come in different sections and for all the variables separately.

The corpus contains 160 dissertations written by both male and female PhD candidates, both in hard and soft sciences, from which, 40 ones were written by non-native Iranian PhD students, and the rest, i.e., 120, were written by natives. Meanwhile, 40 abstracts, out of 120, were taken from hard science fields and only from male writers as we were not able to find an equal number of hard science abstracts from female writers. The other 80 abstracts were chosen from soft science fields by both male and female writers.

### 4.1 Cross-culture variations

**Fig. 2:**
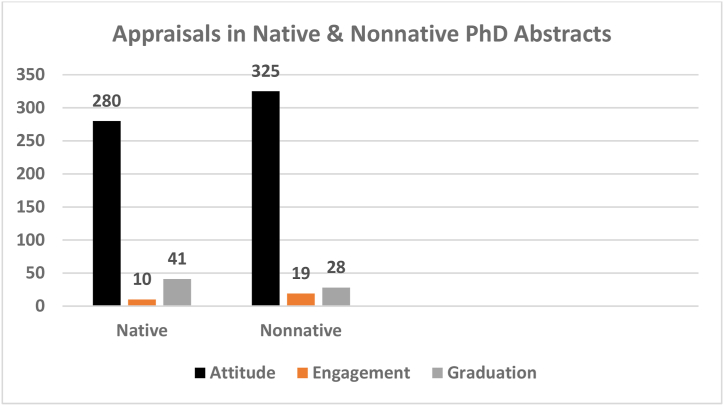
Appraisal resources in native and nonnative.

40 native and 40 nonnative PhD dissertation abstracts, both from soft sciences, form the basis of the analysis for the cross-cultural variations. The reason for selecting only from soft science fields is the unavailability of the nonnative hard science dissertation abstracts. Below, we set forth the results and discussions for the analysis of native and nonnative abstracts for each of the Appraisal categories and subcategories. Fig. 2 presents the results.

#### 4.1.1 Attitude

Attitude is those language resources by which the writers or speakers attempt to express their attitudes towards people, entities, and events. It is divided to Affect, Judgment, and Appreciation.

Attitude resources are the most frequently used resources in the abstracts, making up roughly 85% of the entire resources, and the writers, both native and nonnative, therefore, have been more attentive to expressing their attitudes and evaluations than any other things. They, however, have prolifically used Appreciation resources to the exclusion of the other resources, i.e., Affect and Judgment. The analysis of the data revealed that neither of the two groups (natives & nonnatives) have used Affect and Judgment resources (Fig. 2). This might be out of the ordinary as no emotional reactions and judgments have been made by the writers. In case of Affect, of course, it is much expected as academic and scientific research has no room for emotions and feelings due to the objectivity and impersonality of its nature. They have avoided judging others as this is, too, far from being academic and scientific as objects, processes, and events are the subjects of science rather than people.

Appreciation and, specifically, *evaluation* are the most prominent features in the selected abstracts and the writers evaluate and comment more and more on academic issues they present which is, to the researcher, quite reasonable and natural. However, native English writers have used much more evaluation than nonnative Iranian writers. While native writers have also used more criticism, nonnative writers attempted to describe and explain and less to criticize their colleagues. Thus, it could be interpreted that native writers are more straightforward and critical in tone, but nonnative writers tend to describe and sometimes justify. Another observation made here is the indirect and impersonal criticism leveled in the abstracts which is in conflict to the direct way of nonnative style of criticism used by Iranian writers.

The analysis of the abstracts also revealed that nonnative writers used an objective and impersonal style of presentation as compared to native writers, that is, although all the abstracts were authored by one writer, they used no cases of first-person singular pronoun and favored first person plural pronoun, if applied. Native writers, however, used first person singular pronoun and the number of writers defined the decision.

#### 4.1.2 Engagement

Engagement is reminiscent of Bakhtin's perception of dialogism. It is a set of resources with which the writer indicates his voice in regard to the viewpoints being presented and the probable reactions to these views. Engagement provides the writer or speaker with some resources to obviously convey, exchange and assume explicit inter-subjective and ultimately conceptual stances. Engagement is attending readers' positions and inviting them to follow the issues presented by appealing to other orientations and collective knowledge to present a new view or a new side of the same view which is the dialogic part of Engagement. In Martin and White's [5] words, the writer recognizes the prior standings about the point in a way that the propositions can be recognized as standing with, against, undecided, or as neutral.

In terms of frequency, Engagement resources are the least used resources in PhD abstracts by both native and non-native writers (Fig. 2). These writers, therefore, showed little attention to other positions and did not offer sufficient background in terms of other related voices which is the expansion part of Engagement. They, therefore, failed to make sufficient references to other positions which deepens the abstracts and makes more information available to the readers with regard to the issues being discussed. Nonnative writers, however, in comparison to native writers used more Engagement resources which is indicative of their attentiveness more to other viewpoints and positions.

The Engagement resources used in these PhD abstracts, as Pascual & Unger [24] observe, are chiefly *expansive* meaning that these authors show an aptness for inviting rather than challenging others’ viewpoints and, thus, indicate their openness to other prospects than merely offering their own views which, in this case, shows nonnative priority for these resources more than native ones. The conclusion could be drawn that they try to deal possibly with diverse audience. The result offer assistance to writers by suggesting to paying due attention to interpersonal resources employed to position their stance and induce the readers to support their arguments.

#### 4.1.3 Graduation

Graduation is the last Appraisal element. Graduation, containing the scaling measures of Force and Focus, is the grading phenomena, whereby feelings are amplified and categories blurred [6]. Graduation resources hold the middle position in the frequency of the resources used in the PhD abstracts by both native and non-native writers. Considering Graduation, unlike Engagement, native writers have been, to some extent, more prolific than nonnative writers (Fig. 2). As these resources can create a better impression, bring about a stronger meaning communication, and an influential voice, therefore, native PhD abstract writers are more expressive, inspiring and, powerful in creating the desired impact.

### 4.2 Cross-gender variations

**Fig. 3:**
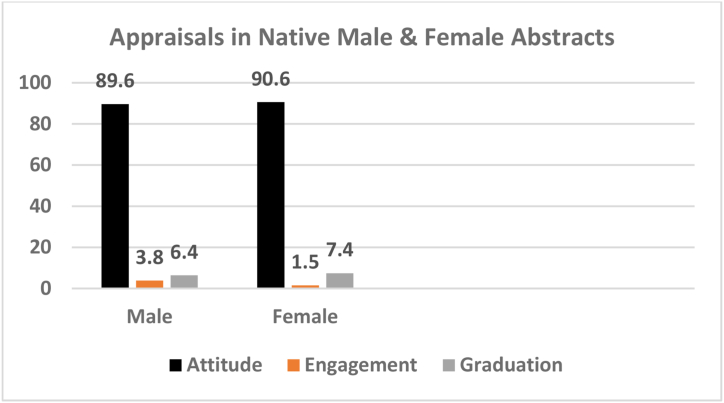
Appraisal resources in native male and female abstracts.

An important point in academic writing is the issue of male and female style of creation and authorship which is rooted in their mentality, individual differences, orientations, and social and cognitive background, and are demonstrated in the structure, rhetorical organization, various idiosyncratic tools and instruments which realize these variations. Appraisal resources, too, are one important aspects of such differences that assist us discover gender-related issues in creating PhD dissertation abstracts. These differences, in terms of Appraisal resources, are discussed in the following paragraphs for each Appraisal category. Fig. 3 shows the results.

#### 4.2.1 Attitude

Attitude, which is classified into Affect, Judgment, and Appreciation, is those language resources by which the writers or speakers attempt to express their attitudes towards people, entities, and events. Attitude enjoys the most frequency and is the most extensive of the whole resources in both native male and female abstracts, making up roughly over 90% of the entire resources. The writers, both male and female, thus, have been more attentive to expressing their attitudes and evaluations than any other things (Fig. 3).

They, however, have profusely used Appreciation resources to the exclusion of the other resources, i.e., Affect and Judgment. The analysis of the data revealed that neither of the two groups (male & female) attempted Affect and Judgment resources. This might be interpreted as an ordinary and regular case scientific writing has no place for the expression of emotional reactions and affective feelings by the writers. In case of Affect, of course, it is much expected as academic research is not subjective due to the objectivity and impersonality of its nature. They have avoided judging others as this is, too, far from being scientific as objects, processes, and events are the subjects of science rather than people.

Another observation is made here concerns the number of Appreciation resources in male and female abstracts. It is noteworthy that male writers, in general, have a superior performance in the use of such resources that might be indicative of their greater verbal skill compared to female writers. This could be surprising, to the researcher, that, opposite the common belief held by many, it is not the female group who is better in this case, but the male camp.

The analysis of the data reveals another interesting finding which entails the polarity, positivity and negativity, of the Appreciation resources used by male and female PhD writers. The results indicate that, although the resources used are extremely positively loaded, male writers used more negative resources compared to female writers. They also were not hesitant to show their negative evaluations explicitly. Thus, the conclusion might be drawn that native male writers are more pessimist in their evaluations, as females are more optimist, and also more straightforward in using explicit negative evaluation resources.

#### 4.2.2 Engagement

In terms of Engagement, both male and female writers used the least number of these resources in PhD abstracts by both native male and female writers (Fig. 3). These writers, therefore, ignored other positions and did not base the abstracts on adequate background of other related voices which is the expansion part of Engagement. They, therefore, failed to provide sufficient conceptual foundation, in terms of other positions, to develop the abstracts and make more information available to the readers. Native writers, however, in comparison to female writers used massively much more Engagement resources (3 times more) which is indicative of their greater attentiveness to other viewpoints and positions. Male writers, thus, are more concerned with others’ standings and views in their writings. In addition, these resources are chiefly expansive (Entertain and Attribute) than contractive.

#### 4.2.3 Graduation

Graduation is the last Appraisal element. Graduation, containing the scaling measures of Force and Focus, is the grading phenomena, to amplify the feelings and blur categories [5]. Graduation resources hold the middle position in the frequency of the resources used in the PhD abstracts by both native male and female writers. Considering Graduation, unlike Engagement, native female writers have been, to some extent, more active than male writers (Fig. 3). As these resources can create an effective impression, bring about strength in communication, and a persuasive voice, therefore, native female PhD abstract writers are more expressive and inspiring in creating the desired impact.

### 4.3 Cross-disciplinary variations

**Fig. 4:**
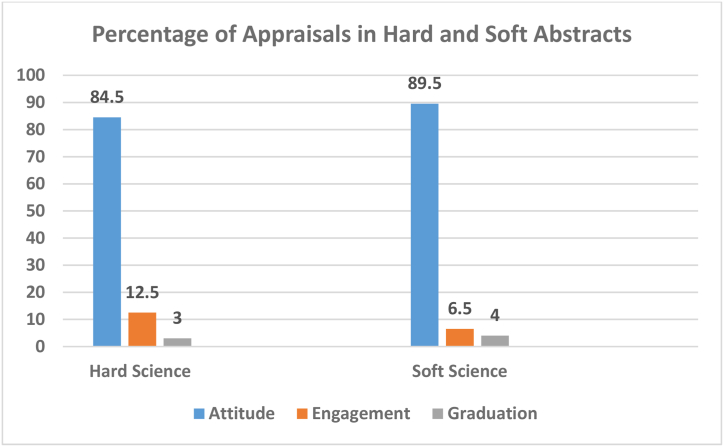
Percentage of Appraisal resources in hard and soft science.

Every discipline is particular in its theme, representation, requirements, and procedures, and the Appraisal resources are good instruments to serve the good purpose of indicating these particularities and materialize the differences. In this study, we have 40 PhD abstracts from the soft science fields such as literature, sociology, linguistics, art, and psychology, and another 40 from hard science majors including mathematics and physics, both by native writers. The differences in terms of Appraisal resources usage are presented for each Appraisal category in the following paragraphs. Fig. 4 displays the results.

#### 4.3.1 Attitude

Attitude functions to express emotional reactions, judgments, and negotiating feelings with response to people, entities, and events. Just like the cases presented earlier, Attitude is the most frequent and extensive category of resources in both hard and soft science PhD abstracts. This class of the Appraisal framework constitutes, and there is a tremendous wealth of Attitude resources nearly 87% of the whole resources. In other words, both hard and soft science PhD writers were more careful to express their attitudes and evaluations to the readers.

Amongst the three Attitude subcategories, native hard and soft science writers in the study, however, have abundantly used Appreciation resources to the exclusion of the other resources, i.e., Affect and Judgment. The results of the data analysis indicated that the soft group did not attempt Affect and Judgment resources at all, but the hard science writers used only two cases which is not significant. This is regarded as a common happening as scientific writing avoids the expression of emotional reactions and affective feelings. In case of Affect, naturally, it is much predictable as academic research is not subjective due to the neutrality and impersonality of its nature. They both have evaded judging others as this is, too, distant from being scientific as objects, processes, and events are the focus of science rather than people. Interestingly, however, the hard group, despite the much stricter nature of their field, used a limited number of these resources (Fig. 4).

Another variation observed here involves the frequency of Appreciation resources in hard and soft abstracts. The soft science group, compared to the hard group, had considerably a better performance and, overall, used more Appreciation resources (2.5%) which might indicate their greater verbal skill and reasoning powers.

The results of data analysis also led to another interesting discovery which entails the polarity, positivity and negativity, and explicitness of the Appreciation resources used by the hard and soft writers. It was revealed that, the resources in spite of being extremely positively loaded, hard writers used 2.5% more negative resources as compared to soft writers. We, therefore, can conclude that native hard writers are, to a limited extent, more pessimist in their attitudes and evaluations as the soft ones are more optimist. Meanwhile, they also used, overtly, more explicit negative attitudes than the soft science writers. Fig. 4 displays the point.

#### 4.3.2 Engagement

Engagement resources are the least frequently used resources in the analyzed PhD abstracts by both hard and soft writers (Fig. 4), and they, thus, unnoticed others' prospects and did not attempt to build adequate context of other related voices, in the abstracts, which is the expansion part of Engagement. They, therefore, were not successful in creating a satisfactory conceptual basis, in terms of other views, to advance the abstracts, justify their choice, and make available more information on different aspects of the issue to the readers. The soft writers, however, in comparison to the hard group, used a little more Engagement resources which points out to their, somewhat, attention to other views and stances. Male writers, thus, more care about others’ standings and views. In addition, these resources are chiefly expansive (containing Entertain and Attribute) than contractive.

#### 4.3.3 Graduation

Graduation, the final Appraisal element, comprises the scaling measures of Force and Focus, is the grading phenomena, to amplify the feelings and blur categories [6]. Graduation resources hold the middle place in the frequency of the resources used in the PhD abstracts by both hard and soft science writers.

With reference to Graduation, in contrast to Engagement, native hard writers have been, profoundly, more active than soft writers using two times more resources (Fig. 4). As these resources assist in constructing an efficient impression, add force to communication, and a convincing voice, therefore, native hard PhD abstract writers are more expressive and inspiring in creating the desired impact. This is quite unordinary and opposite to the common expectations; the soft science writers are more expected to have a more powerful writing style due to the subjectivity of their discipline as compared to the strict and austere nature of hard science field.

### 4.4 Interpretation of the findings in relation to gender, language, culture and discipline

#### 4.4.1 Gender variations

• **Attitude Resources**: The study reveals that all writers, regardless of gender, extensively employ Attitude resources to convey their stances and evaluations. However, there may be nuances in how different genders express their attitudes. Further analysis is required to understand if there are statistically significant gender-based differences in the use of specific Attitude resources, such as Appreciation, Affect, or Judgment.
• **Appreciation Resources**: Female soft science writers stand out as the most proficient in using Appreciation resources. This suggests that they excel in conveying their emotional and evaluative stances, making them more persuasive and inspiring in their abstracts. Future research could explore why this gender difference exists and whether it extends to other academic writing contexts.
• **Graduation Resources**: Graduation resources are used with a minor difference across genders. However, it would be beneficial to investigate if any particular aspects of Graduation, such as Force or Quantity, exhibit gender-based variations that could provide deeper insights into writing preferences and strategies.
• **Engagement Resources**: Engagement resources are the least frequently employed by all writers. This suggests a common trend among both male and female writers, irrespective of their gender or field of study. Further research could explore why these resources are underutilized and whether their inclusion would enhance the quality and persuasiveness of the abstracts.

#### 4.4.2 Language variations

• **Attitude Resources**: The study does not specifically mention language-based variations in the use of Attitude resources. Future analyses could explore whether non-native writers differ significantly from native English writers in expressing their attitudes in abstracts.
• **Appreciation Resources**: The superior performance of female soft science writers in using Appreciation resources raises questions about whether language proficiency influences this aspect of Appraisal. Further analysis may reveal whether non-native writers face challenges or exhibit variations in their use of Appreciation resources.
• **Graduation Resources**: The minor difference in the use of Graduation resources may not be language-dependent. However, it would be valuable to examine if language proficiency impacts the application of specific Graduation resources, such as Force or Quantity.
• **Engagement Resources**: The underutilization of Engagement resources is not specifically attributed to language differences. Nonetheless, future research could explore whether non-native writers face language-related obstacles in presenting other positions and prospects in their abstracts.

#### 4.4.3 Cultural variations

• **Attitude Resources**: The study highlights Attitude resources as dominantly employed across cultures, suggesting that this linguistic aspect transcends cultural boundaries. However, further analysis may uncover subtle cultural influences on how Attitude resources are utilized.
• **Appreciation Resources**: The superior performance of female soft science writers in using Appreciation resources may be indicative of cultural nuances in communication styles or preferences. Future research could explore whether cultural factors contribute to this variation.
• **Graduation Resources**: The study does not explicitly address cultural variations in Graduation resource usage. Investigating whether cultural norms affect the use of specific Graduation resources could provide valuable insights.
• **Engagement Resources**: The underutilization of Engagement resources is not attributed to cultural differences in the study. Future research could delve into how cultural perspectives impact the inclusion of alternative viewpoints in abstracts.
4. Discipline Variations
• **Attitude Resources**: The prevalence of Attitude resources is consistent across both hard and soft science fields. However, it would be interesting to explore whether specific disciplines exhibit distinct preferences for certain Attitude resources, reflecting their unique communication styles.
• **Appreciation Resources**: The superior performance of female soft science writers in using Appreciation resources suggests potential discipline-related variations. Research could investigate if the impact of gender on Appreciation resource usage is consistent across different academic disciplines.
• **Graduation Resources**: Graduation resources, especially related to Force, are more frequent. Further analysis might reveal if specific hard or soft science fields demonstrate distinct preferences within Graduation resources.
• **Engagement Resources**: Engagement resources are underutilized across disciplines. Examining whether certain fields, such as philosophy or psychology, employ Engagement resources more frequently could provide valuable insights into disciplinary writing conventions.

## 5 Conclusion, implication, and application

This is an extensive study on the use of Appraisal resources in PhD dissertation abstracts. In the current study, we employed Martin & White's [5] Appraisal Framework to explore the cross-culture, cross-gender, and cross-disciplinary differences in using Appraisal resources in native and nonnative male and female PhD dissertation abstracts. The corpus included 160 abstracts written by native and nonnative male and female PhD candidates, both in hard sciences (mathematics and physics) and soft sciences (philosophy, art, literature, psychology, and linguistics).

The findings of the research indicate that all writers, in this study, abundantly used appraisal resources to express their stances, prospects, perceptions, and evaluations of various topics and problems to the readers. A glance at the tables and figures, however, revealed the foremost supremacy of the **Attitude** resources in all PhD abstracts and all the abstracts, thus, are attitudinally dominated, that is, Attitude resources per se are two time more than the other two Appraisal groups of resources together. These resources constitute, overall, 84% of the total Appraisal resources used in the all abstracts, whether native or nonnative, male or female, and hard science or soft science fields.

Concerning Appreciation resources, female soft science writers, relatively, had a better performance and outperformed all the other writers. Female soft science writers, therefore, are the most ingenious writers, based on this study, and have greater interpersonal communication power, and, hence, more inspiring and persuasive. However, more studies are needed to generalize the findings across the disciplines.

Graduation resources, although with a trivial difference, occupy the middle position in the application of Appraisal resources. They make up 8.9% of the whole resources in the all abstracts which is so much less than the frequency of Attitude resources mentioned above. Within Graduation, resources related to Force are the most frequent resources and enjoy greater popularity in all the abstracts.

Engagement resources, with a fragile difference with Graduation, form 7.1% of the Appraisals, are the least frequently used resources in the selected abstracts. These resources are concerned with presenting other positions and prospects related to the topic of inquiry. They, consequently, were less prosperous in building a reasonable conceptual basis, in terms of other views, to progress the abstracts, substantiate their positions, and make available more information on different aspects of the issue to the readers. Within Engagement, Heteroglossic resources containing *expansive (entertain* and *attribute)*, encompassing resources are much more common than the *contractive* ones.

This study has direct bearing on teaching and learning. In terms of teaching, we advise that the Appraisal resources be integrated into the development of EFL and ESL teaching materials, course books, and tasks. Incorporating these resources into language teaching curriculum and introductory writing programs can pave the way for instilling a better writing skill, evaluation, and critical thinking in learners. Writers and speakers, too, can assist readers and learners and enhance their comprehension with an appropriate and active use of Appraisal resources.

In terms of learning, it is recommended that EFL and ESL learners focus on Appraisal resources as effective instruments of developing and improving writing ability and keeping up with the necessities of academic writing and, especially, writing research papers and articles. Using these resources, learners are well equipped with the tools to express their attitudes, create their own style, and critically interpret other authors compositions. Familiarity and a sound knowledge of the Appraisals, consequently, can be a constructive tool and lead to the expansion of general writing skills which is considered as a main concern in novice writers.

Implementing appraisal resources into teaching materials in a corporation can greatly enhance the learning experience for employees. As the writer suggested the incorporation of appraisal resources into the teaching and learning material, to ensure effective implementation, in the following I provide some guidance steps to follow.

• Identify the Learning Objectives: Begin by clarifying the learning objectives for the teaching materials. Determine what specific skills, knowledge, or competencies the employees should acquire through the appraisal resources.
• Select Appropriate Appraisal Resources: Choose appraisal resources that align with the learning objectives and are relevant to the employees' roles and responsibilities. Examples may include case studies, real-life scenarios, self-assessment tools, performance evaluation templates, or feedback models.
• Integrate Appraisal Resources: Incorporate the selected appraisal resources into the teaching materials. This can be done by creating dedicated sections within the materials, embedding relevant examples or exercises, or providing links to external resources.
• Contextualize the Materials: Ensure that the teaching materials provide clear explanations of how the appraisal resources relate to the employees' work context. Help learners understand how the concepts and techniques can be applied in their day-to-day tasks, projects, or interactions.
• Provide Guidance on Appraisal Process: If the appraisal resources involve specific processes or methodologies, provide step-by-step guidance on how to use them effectively. Break down complex concepts, highlight critical considerations, and provide practical tips or best practices.
• Encourage Active Learning: Design the teaching materials to foster active learning. Include interactive elements such as quizzes, case discussions, group activities, or role-playing exercises that encourage employees to apply the appraisal resources in simulated or real-world scenarios.
• Offer Supportive Materials: Supplement the teaching materials with additional resources that support the appraisal process. These could include job aids, reference guides, video tutorials, or templates that employees can access as they implement the appraisal resources.
• Promote Discussion and Collaboration: Encourage employees to engage in discussions and collaborate with their peers during the learning process. This can be facilitated through in-person or virtual meetings, forums, or online platforms, allowing participants to share experiences, insights, and challenges related to the appraisal resources.
• Provide Ongoing Feedback and Support: Continuously provide feedback to employees as they engage with the teaching materials and implement the appraisal resources. Offer support through mentoring, coaching, or follow-up sessions to address questions, clarify doubts, and provide guidance as needed.
• Evaluate Learning Outcomes: Assess the success of the implemented appraisal resources by evaluating the learning outcomes. Collect feedback from employees, measure their performance improvements, and use the insights gained to refine and enhance the teaching materials for future use.

## Data availability statement

All the texts analyzed in the present study have been selected from sources which are available online.

## CRediT authorship contribution statement

**Ali Hashemi:** Writing – review & editing, Writing – original draft, Validation, Resources, Project administration, Methodology, Investigation, Formal analysis, Conceptualization. **Fatemeh Mahdavirad:** Writing – review & editing, Writing – original draft, Validation, Supervision, Resources, Project administration, Methodology, Investigation, Formal analysis, Conceptualization.

## Declaration of competing interest

The authors declare that they have no known competing financial interests or personal relationships that could have appeared to influence the work reported in this paper.
